# Performance Comparison of U-Net and Its Variants for Carotid Intima–Media Segmentation in Ultrasound Images

**DOI:** 10.3390/diagnostics16010002

**Published:** 2025-12-19

**Authors:** Seungju Jeong, Minjeong Park, Sumin Jeong, Dong Chan Park

**Affiliations:** 1 Division of Artificial Intelligence Engineering, Korea Maritime and Ocean University, Busan 49112, Republic of Korea; wjdtmd2323@g.kmou.ac.kr (S.J.); popop0078@g.kmou.ac.kr (M.P.); 2 Department of Electronic and Electrical Engineering, Department of IT Convergence Engineering, Kumoh National Institute of Technology, Gumi-si 39177, Gyeongsangbuk-do, Republic of Korea

**Keywords:** cardiovascular diseases, atherosclerosis, carotid intima-media thickness, ultrasound image segmentation, deep learning, U-Net, real-time efficiency

## Abstract

**Background/Objectives**: This study systematically compared the performance of U-Net and variants for automatic analysis of carotid intima-media thickness (CIMT) in ultrasound images, focusing on segmentation accuracy and real-time efficiency. **Methods**: Ten models were trained and evaluated using a publicly available Carotid Ultrasound Boundary Study (CUBS) dataset (2176 images from 1088 subjects). Images were preprocessed using histogram-based smoothing and resized to a resolution of 256 × 256 pixels. Model training was conducted using identical hyperparameters (50 epochs, batch size 8, Adam optimizer with a learning rate of 1 × 10^−4^, and binary cross-entropy loss). Segmentation accuracy was assessed using Dice, Intersection over Union (IoU), Precision, Recall, and Accuracy metrics, while real-time performance was evaluated based on training/inference times and the model parameter counts. **Results**: All models achieved high accuracy, with Dice/IoU scores above 0.80/0.67. Attention U-Net achieved the highest segmentation accuracy, while UNeXt demonstrated the fastest training/inference speeds (approximately 420,000 parameters). Qualitatively, UNet++ produced smooth and natural boundaries, highlighting its strength in boundary reconstruction. Additionally, the relationship between the model parameter count and Dice performance was visualized to illustrate the tradeoff between accuracy and efficiency. **Conclusions**: This study provides a quantitative/qualitative evaluation of the accuracy, efficiency, and boundary reconstruction characteristics of U-Net-based models for CIMT segmentation, offering guidance for model selection according to clinical requirements (accuracy vs. real-time performance).

## 1. Introduction

The CIMT is an important indicator of atherosclerosis progression and the risk of future cardiovascular events, including stroke and myocardial infarction. Elevated CIMT is directly associated with increased vascular stiffness and plaque burden, making its accurate measurement essential for patient risk stratification and monitoring therapeutic effectiveness [1]. CIMT can be assessed using various non-invasive medical imaging modalities. While magnetic resonance imaging provides excellent soft tissue contrast, its high cost and limited accessibility restrict its routine clinical use. Computed tomography offers detailed visualization but involves exposure to ionizing radiation. For routine clinical practice, B-mode ultrasound remains the gold standard. It offers a cost-effective, real-time, and safe method to visualize the carotid artery wall structure. To ensure standardized assessment and reliability, international consensus guidelines such as the Mannheim Consensus recommend measuring CIMT on the far wall of the common carotid artery (CCA) over the straight segment located approximately 10 to 30 mm proximal to the carotid bulb [2].

Despite the advantages of ultrasound imaging, manual CIMT measurement is inherently time-consuming, tedious, and highly prone to inter- and intra-observer variability. These factors significantly limit its utility in large-scale clinical trials and routine practice. To overcome these limitations, automated segmentation techniques leveraging artificial intelligence have been extensively studied. In particular, deep learning has emerged as the leading approach for precise and robust medical image analysis. U-Net has become a standard model for medical image segmentation [3,4,5,6]. Subsequently, numerous variants, including ResUNet, UNet++, Attention U-Net, and the transformer-based TransUNet, have been proposed and have demonstrated significant performance improvements across diverse tasks [7,8,9,10,11].

Recent studies have applied U-Net variants to CIMT or carotid ultrasound image segmentation. For example, Ottakath et al. proposed a framework termed *Refined Intima-Media Segmentation* to improve boundary reconstruction performance [12]. Jain et al. successfully segmented pathological regions, including plaques, using an attention-based U-Net [13]. In addition, Al Qurri and Almekkawy introduced a novel attention module that outperformed vanilla U-Net across multiple datasets [14]. Further, Biswas et al. compared traditional techniques with neural-network-based methods, highlighting both the potential and limitations of automated CIMT measurement [15]. More recently, Tian et al. quantitatively analyzed the radial arterial tracking performance in the short-axis view by comparing several U-Net family models [16].

However, despite these advances, many comparative studies have not yet jointly examined critical aspects, such as boundary reconstruction quality, real-time inference speed, and model parameter counts. Furthermore, direct head-to-head evaluation of the U-Net family models on CIMT ultrasound images remains limited. In this study, we quantitatively compared the performance of U-Net and its variants using a publicly available carotid ultrasound dataset in which all models were trained and tested under an identical experimental protocol. The evaluation metrics included Dice/IoU scores, precision, recall, pixel-wise accuracy, training time, inference speed, and parameter counts. Through this, we aimed to provide a comprehensive characterization of each model, thereby clarifying the relative strengths and limitations of automated CIMT measurements.

### 1.1. Basic U-Net Architecture

#### 1.1.1. Overview of the Basic U-Net Architecture [3]

U-Net has a U-shaped symmetric structure composed only of convolutional layers consisting of a contracting path on the left and an expansive path on the right. U-Net was designed based on the concepts of double convolutional blocks, an encoder, a decoder, and skip connections. With this configuration, U-Net can effectively perform the pixel-wise segmentation of input images and has shown excellent performance, particularly in the medical imaging field. In addition, to address the lack of large training datasets for medical imaging problems, effective network training with a small number of images was devised by leveraging data augmentation.

#### 1.1.2. Encoder–Decoder Structure

The encoder functions as a contracting path based on convolutional neural networks (CNNs), which utilize learnable filters (kernels) to capture hierarchical spatial patterns—ranging from low-level edges to high-level semantic features. The encoder extracts these features from the input image via convolution operations and pooling, while gradually reducing the spatial resolution. Each encoder stage applies a double convolutional block consisting of two 3 × 3 convolution layers, followed by a 2 × 2 max-pooling operation that halved the height and width of the feature maps. With each downsampling step through pooling, the number of channels (feature maps) doubles, and richer representations are learned. The features hierarchically extracted by the encoder from low-to high-level representations are stored such that they can be used in the decoder stage.

Conversely, the decoder proceeds in the opposite direction to the encoder, progressively restoring the low-resolution feature maps to output a segmentation result of the same size as the original input. At each decoder stage, features are upsampled to double the resolution and concatenated with the skip connection features stored from the corresponding encoder stage. A double convolutional block is then applied again to the concatenated features so that the interactions among the combined information can restore the fine details necessary for segmentation. At each decoder stage, the number of channels is halved, reversing encoder doubling, such that only as many channels as the number of target classes remain in the final output. Finally, a 1 × 1 convolution layer produced the final per-pixel class probabilities or labels to obtain the segmentation map.

#### 1.1.3. Skip Connection

The skip connections of U-Net directly deliver high-resolution feature maps extracted from the encoder to the decoder. The feature maps obtained immediately before pooling at each encoder stage are stored and later concatenated with the upsampled feature maps at the corresponding decoder stage, thereby integrating fine details from the original image with high-level semantic information. Through these skip connections, multiresolution features can be effectively fused so that delicate parts, such as segmentation boundaries, can be accurately reconstructed. In other words, skip connections compensate for the localization information lost in deep networks to achieve high localization accuracy.

### 1.2. Overview of U-Net Variants

To improve the segmentation accuracy in medical imaging, various variants have been proposed to enhance the basic U-Net structure. Representative examples include ResUNet, which introduces residual connections to enable the training of deeper networks, and R2U-Net, which improves performance with efficient parameters using a recurrent residual structure [15]. UNet++ provides richer feature information through hierarchical and dense connections [17], whereas Attention U-Net introduces an attention mechanism that selectively focuses on important regions [18]. More recently, Transformer-based TransUNet addresses long-range dependency issues and has gained attention [12]; UNext combines convolution and MLP to achieve a lightweight model while maintaining high performance [13]. The structural characteristics and performance-enhancing elements of these variants are examined in the following subsections.

#### 1.2.1. ResUNet [11]

ResUNet extends the basic U-Net model by introducing residual connections, replacing U-Net’s convolutional blocks with residual blocks, which add the block input to its output as a skip connection. These residual connections facilitate gradient flow during training, mitigate vanishing gradients in deep networks, and enable the training of deeper architectures. By combining U-Net’s strong segmentation capacity with ResNet’s stable training characteristics, ResUNet can effectively capture fine image details and improve the convergence and segmentation performance.

#### 1.2.2. UNet++ [7,8]

UNet++, also known as a Nested U-Net, is a model that hierarchically redesigns the skip connections of U-Net. While the original U-Net directly connects features at the same resolution between the encoder and decoder, UNet++ inserts additional convolutional layers between them, forming multistage nested skip pathways. This nested dense skip pathway gradually reduces the semantic gap between the encoder and decoder, effectively fusing features across multiple resolutions. In actual experiments, UNet++ has demonstrated an average IoU improvement of approximately 3.9 points over the basic U-Net, validating the effectiveness of this structural refinement.

#### 1.2.3. Attention U-Net [9]

Attention U-Net introduces attention gates into the skip connections of U-Net. Using a gating signal that combines features propagated from the encoder with contextual information on the decoder side, this model assigns higher weights to important spatial regions in the feature maps to pass through skip connections, while suppressing irrelevant parts. This attention mechanism enables better discrimination between target structures and backgrounds, thereby improving segmentation accuracy, even for small structures and complex scenes.

#### 1.2.4. TransUNet [10]

TransUNet is a hybrid model that integrates Transformers into the U-Net architecture to compensate for the locality limitations of the convolution-based U-Net. In this model, a Vision Transformer is introduced into the encoder, and the input image is tokenized into patches and processed using a self-attention mechanism to extract global contextual information. The extracted global feature representation is fused with high-resolution CNN feature maps via skip connections in the decoder to restore the fine spatial information. With this design, TransUNet has shown outstanding accuracy, surpassing existing CNN-based models in various medical imaging tasks.

#### 1.2.5. UNext [19]

UNext is a lightweight convolution–MLP hybrid U-Net proposed for fast medical image segmentation. The early encoder used convolution layers to extract features; in the latent space, the feature maps were tokenized and processed by MLP blocks, performing MLP-based representation learning instead of conventional convolutions. This design makes the model highly lightweight, reducing parameter counts to approximately 1/72 and computations to approximately 1/68 of traditional models, while maintaining high accuracy.

#### 1.2.6. Inception U-Net [20,21]

Inception U-Net applies inception modules to the encoder and decoder of a basic U-Net to simultaneously extract features at multiple scales. Each Inception module performs 1 × 1, 3 × 3, and 5 × 5 convolutions, with a pooling path in parallel, and concatenates the results along the channel dimension to provide rich multiscale information. The 1 × 1 convolution reduces the dimensionality before the larger-kernel convolutions to reduce computation, after which the large-kernel convolutions capture wide-receptive-field information. This structure can effectively capture both small and large lesions, thereby improving the medical image segmentation performance.

#### 1.2.7. Attention Res-U-Net [22]

The attention Res-U-Net combines attention mechanisms with the Res-U-Net architecture to selectively focus on important regions. Using an attention block that combines a gating signal and an input feature map, attention weights that indicate the importance of each spatial location are computed. The computed weights were applied element-wise to the feature maps that passed through skip connections, suppressing unnecessary information and emphasizing meaningful regions.

#### 1.2.8. SE U-Net [23,24]

SE U-Net integrates squeeze-and-excitation (SE) blocks into the basic U-Net structure to strengthen its ability to learn interchannel dependencies. In the squeeze stage, the global average pooling compresses the spatial information of each channel into a single value to form a channel-wise statistical vector. In the excitation stage, this statistical vector is used to learn channel-wise weights, thereby enabling the network to emphasize important channels and suppress unnecessary ones.

#### 1.2.9. Dense U-Net [25,26]

Dense U-Net incorporates DenseNet’s dense and transition blocks within the U-Net structure to reinforce feature reuse and compensate for resolution loss. The network comprises a symmetric dense downsampling path and a dense upsampling path. Within each dense block, every layer is connected to all of the previous layers to maximize feature utilization. Each dense block comprises BN → 1 × 1 Conv (channel reduction) → 3 × 3 Conv (feature extraction) → Dropout with four densely connected layers. Transition blocks are composed of BN → 1 × 1 Conv → 2 × 2 Max Pooling to perform inter-stage transitions and resolution reduction. In the upsampling path, the merge operations and dense blocks restore the resolution and ultimately produce a full-resolution output.

## 2. Materials and Methods

### 2.1. Dataset and Preprocessing

For the multi-institutional analysis, we used the CUBS dataset publicly available on Mendeley Data [27]. CUBS included far-wall segments of the left and right CCA in B-mode ultrasound images collected from a total of 1088 patients (mean age: 62 ± 11 years; 50% female). The study population comprised two distinct cohorts: 694 participants recruited from the general population in Cyprus (inclusion criterion: age > 40 years) and 394 patients enrolled from a Hypertension Outpatient Clinic in Pisa, representing a group with higher cardiovascular risk factors. The dataset provides up to two images per patient (yielding 2176 total frames), along with segmentation masks manually annotated by three experts. Ultrasound scans were performed using a Philips ATL HDI-5000 duplex scanner with an L12-5 MHz linear array probe (1388 images from 694 patients in Cyprus) and an Esaote MyLab25 system with an LA523 4–13 MHz linear probe (788 images from 394 patients in Pisa), following the Mannheim consensus guidelines for standardized CCA image acquisition [28]. Among the three experts, the annotations from Analyst A1 (>10 years of clinical experience) were selected as the ground truth (GT), as this expert provided the most complete and consistent annotations across the entire dataset. Regarding data selection, multiple clips were originally acquired, and expert operators selected the single best frame per side based on the highest qualitative visual contrast between the lumen and intima-media complex, thereby excluding images with poor visibility or artifacts that prevented reliable annotation. No further exclusion criteria were applied in our study to maintain the standard benchmark configuration.

The images were provided in TIFF format in 8-bit grayscale, with an average resolution of 720 × 576 pixels. One or multiple frames were provided per patient and the total file size was approximately 378 MB. To address speckle noise and inherently low-contrast vascular boundaries in ultrasound, we applied contrast-limited adaptive histogram equalization (CLAHE), which has been shown to enhance edge definition while avoiding noise amplification compared with global histogram equalization approaches [29,30]. Subsequently, all images and masks were resized to 256 × 256 pixels, and the pixel values were normalized to the range [0, 1]. For data splitting, we applied a patient-level stratified split into 80% for training (870 patients) and 20% for validation (218 patients) to prevent the same patient images from appearing in both training and validation.

### 2.2. Network Architectures

Detailed architectural specifications and the complete source code for all implemented models are publicly available in our GitHub online repository [31]. All models follow the standard architectural configurations described in Section 1, with minor adjustments to accommodate the specific input resolution of the CUBS dataset. To provide a clear overview of the architectural diversity, we summarize the key characteristics of the implemented U-Net variants in Table 1. Each model modifies the standard U-Net backbone by incorporating distinct mechanisms—such as residual connections, attention gates, transformers, or MLP blocks—to address specific segmentation challenges including boundary delineation, long-range dependencies, and computational efficiency.

The filter configuration in Table 1 specifies the number of filters in each layer in the encoder path. The decoder path generally follows this configuration in reverse order to restore spatial resolution. However, some hybrid models exhibit structural asymmetry to improve efficiency: TransUNet uses a purely CNN-based decoder without Transformer layers, and UNeXt employs simple convolutional upsampling in the decoder, omitting the tokenized MLP blocks present in the encoder. While most networks utilize a standard 3 × 3 kernel size for feature extraction, certain architectures adopt different kernel sizes tailored to their design: DenseUNet employs a 7 × 7 convolution in the initial layer to achieve a larger receptive field, while InceptionUNet utilizes parallel multi-scale kernels (1 × 1, 3 × 3, and 5 × 5). In addition, ResNet-based models and UNeXt incorporate 1 × 1 convolutions for residual connections or pointwise operations to reduce computational cost.

The parameter counts reported in this study may vary depending on the initial number of channels and the network depth used in the model implementation and may differ from those reported in prior publications. However, all models were trained and evaluated under identical settings to ensure fairness in the relative comparisons.

### 2.3. Hyperparameter Settings

All models were trained for 50 epochs with a batch size of 8. We applied the Adam optimization algorithm with an initial learning rate of 1 × 10^−4^. We used binary cross-entropy as the loss function for the per-pixel error:

$$L_{BCE}=\;-\frac{1}{N}\sum_{i=1}^{N}\left[y_{i}\ln\left(\sigma \left(x_{i}\right)\right)+(1-y_{i}\right)ln(1-\sigma \left(x_{i}\right))]$$

where N is the total number of pixels, $x_{i}$ is the model output for the i-th pixel, $y_{i}\in \{0,1\}$ is the ground truth label, and $\sigma \left(x\right)=1/(1+e^{-x})$ is the sigmoid fuction.

### 2.4. Segmentation Performance Metrics

To quantitatively evaluate the accuracy of the segmented regions, we calculated the Precision, Recall, Accuracy, and Dice/IoU scores, where TP, FP, TN, and FN denote the true positives, false positives, true negatives, and false negatives, respectively.

• Precision is the proportion of predictions labeled positive by the model that are actually positive.

$$Precision=\frac{TP}{TP+FP}$$

• Recall is the proportion of actual positive instances that the model correctly predicts as positive.

$$Recall=\frac{TP}{TP+FN}$$

• Accuracy is the proportion of all cases the model classifies correctly:

$$Accuracy=\frac{TP+TN}{TP+TN+FP+FN}$$

• Dice score is defined as twice the size of the intersection of the predicted and ground truth regions divided by the sum of their sizes.

$$Dice\;score=\frac{2\times |A\cap B|}{A+B}=\frac{2TP}{2TP+FP+FN}$$

• IoU is defined as the size of the intersection divided by the size of the union of the two regions (intersection over union). All take values in [0, 1]; higher values indicate better agreement between the prediction and ground truth, with 1 indicating perfect segmentation.

$$IoU=\frac{\left|A\cap B\right|}{\left|A\cup B\right|}=\frac{TP}{TP+FP+FN}$$

### 2.5. Measurement of Training and Inference Time

To compare the computational efficiency across the models, we measured both the training and inference times. The training time was recorded until each epoch was completed and converted into the mean training time per epoch. The inference time was measured on the validation dataset with a batch size fixed at 1 and reported as the average time per single ultrasound image. This allows for the quantitative evaluation of the training efficiency and real-time applicability per model.

### 2.6. Experimental Environment

All experiments were conducted in Python 3.10.16, using the PyTorch 2.5.1 deep-learning framework. The hardware used was a laptop equipped with an RTX 4060 Laptop GPU with 8 GB VRAM (NVIDIA, Santa Clara, CA, USA) and an Ryzen 7 7840HS APU processor (AMD, Santa Clara, CA, USA). We also used CUDA 12.1 and cuDNN 9.1.0 for the GPU-accelerated computation.

## 3. Results

### 3.1. Performance Comparison

Segmentation performance was evaluated using Precision, Recall, Accuracy, Dice and IoU scores, whereas the real-time efficiency was assessed based on parameter counts, training time, mean epoch time, and inference time.

Table 2 shows the number of parameters of the U-Net variants. Most U-Net-family models have approximately 8–9 million parameters, but Attention U-Net has the highest at approximately 31.4 million. Conversely, UNext had only about 420 thousand parameters, confirming its extremely lightweight structure. This shows large differences across the models in terms of computational complexity and resource requirements.

Table 3 summarizes the segmentation performance in terms of Dice and IoU scores. All models showed stable performance with Dice ≥ 0.80 and IoU ≥ 0.67. The attention U-Net achieved the highest performance, and UNet++ and SE U-Net were slightly superior to the basic U-Net. UNext had the lowest scores but maintained a respectable performance relative to its extremely small parameter count.

Table 4 presents comparisons of the segmentation performances in terms of Precision, Recall, and Accuracy. Most models maintained a balance between Precision and Recall, and Accuracy above 98%. The attention U-Net had the highest recall but a relatively lower precision, showing a tendency toward oversegmentation. The other models maintained balanced Precision and Recall, demonstrating stable segmentation performance.

Table 5 presents a comparison of the computational time per model. Most models required approximately 40 s per epoch, whereas Attention U-Net required approximately 72 s, which was the longest. Conversely, UNext was the fastest, at about 32 s per epoch with a total training time of about 27 min; this model also had the shortest inference time. In other words, the Attention U-Net stands out in terms of accuracy, whereas UNext stands out in terms of speed and efficiency.

**Figure 1:**
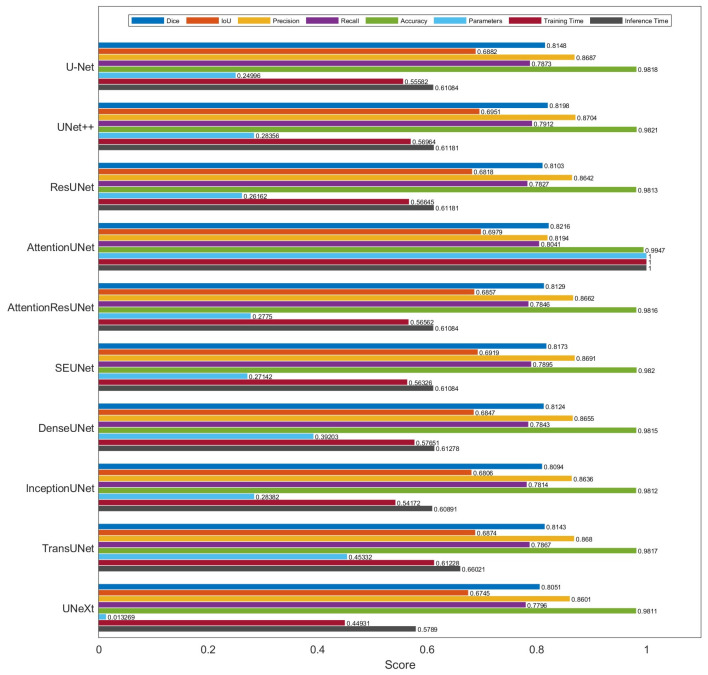
Performance comparison of U-Net variants for CIMT segmentation.

**Figure 2:**
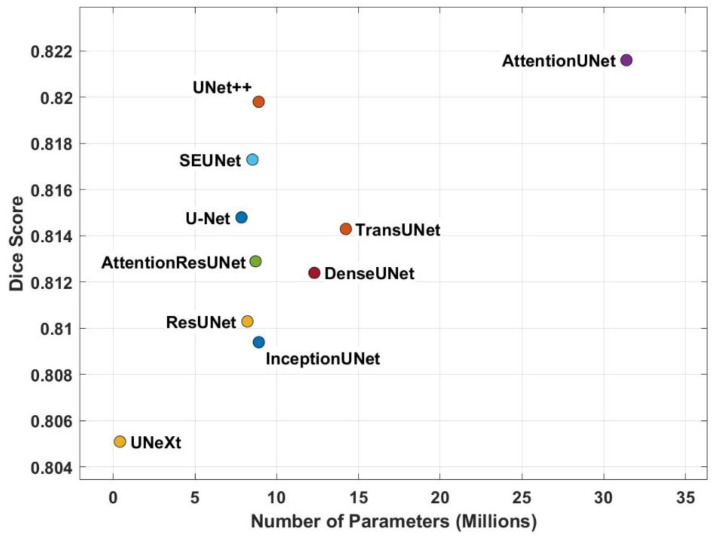
Number of parameters versus Dice score for U-Net variants.

Segmentation performance (Table 3 and Table 4) and the real-time efficiency (Table 2 and Table 5) are summarized in Figure 1. Note that the parameter counts, training time, and inference time are all normalized with respect to Attention U-Net. Figure 2 summarizes the number of parameters and dice scores for several U-Net variants. The results highlight a clear tradeoff between the model complexity and segmentation accuracy. Attention U-Net achieved the highest Dice score (~0.822); however, this gain came at the cost of a markedly larger parameter count (>30 M), implying heavier computational and memory requirements during both training and deployment. In contrast, UNext achieved competitive accuracy with the smallest parameter footprint, suggesting strong potential for resource-constrained or real-time applications, where inference speed and memory efficiency are critical.

Intermediate models, such as UNet++ and SEUNet, demonstrated relatively high Dice scores while maintaining moderate parameter sizes, which may offer a more balanced compromise between accuracy and computational burden. These observations indicate that the optimal model selection should be guided not only by raw segmentation performance but also by practical considerations, such as hardware constraints and deployment scenarios.

### 3.2. Visualization

Because the model outputs were fixed at 256 × 256 pixels, we used bilinear interpolation to upsample them to match the original ultrasound image size (e.g., 720 × 576 pixels). This smoothly enlarges the probability map, thereby allowing for seamless alignment with the original image. In contrast, because the GT mask must retain the binary pixel values (0/1), we applied the nearest-neighbor interpolation. This approach converts the model output and GT appropriately to ensure consistency in visual comparisons.

**Figure 3:**
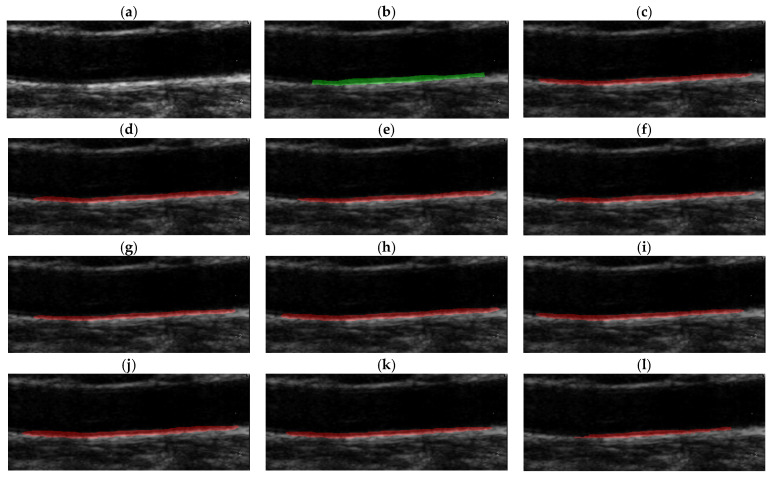
Comparison of CIMT segmentation results using a representative ultrasound image. (**a**) Original image. (**b**) Ground truth. (**c**) U-Net. (**d**) UNet++. (**e**) ResUNet. (**f**) Attention U-Net. (**g**) AttentionResUNet. (**h**) SEUNet. (**i**) DenseUNet. (**j**) InceptionUNet. (**k**) TransUNet. (**l**) Unext. Green denotes the ground truth, while red denotes the segmentation results.

Figure 3 presents the CIMT segmentation results of a representative image. We utilized OpenCV 4.11 for mask generation and applied region filtering to remove small fragments. The U-Net and Attention U-Net generally exhibited stable and respectable performances. U-Net reproduced the overall contours reasonably well, but the boundaries appeared somewhat irregular in areas of high curvature, whereas U-Net captured thin structures relatively well, but showed a tendency towards thicker (over-segmented) boundaries in some cases. Interestingly, UNet++ tended to reconstruct the shape of the vascular lumen more smoothly and naturally than the GT itself, which can be interpreted as compensating for the observer variability inherent in manual GT. Conversely, UNext, despite its extremely small parameter count, reproduced the overall contours well, but showed boundary discontinuities at some endpoints, indicating somewhat limited fine-boundary reconstruction.

## 4. Discussion

Although U-Net variants are widely used in medical image segmentation, systematic comparisons for CIMT boundary segmentation under consistent experimental settings have remained limited. As a result, performance differences reported across studies could not be readily attributed to architectural choices versus dataset- or protocol-specific factors. This study addresses this gap by providing a standardized and reproducible benchmark on a public dataset under uniform training and evaluation settings.

Jain et al. [13] proposed an attention-based U-Net specifically tailored for plaque segmentation in carotid ultrasound images, emphasizing its utility in stroke risk stratification. While their model demonstrated enhanced segmentation of pathological regions, our study focuses on a broader comparison of U-Net variants for CIMT segmentation, where precise boundary delineation for thickness measurement is critical, rather than targeting plaque regions, thereby providing a more generalized evaluation.

Al Qurri and Almekkawy [14] introduced a novel spatial attention mechanism that improved performance across various datasets. In contrast, we implemented multiple established variants including Attention U-Net as part of a comprehensive benchmark under uniform training and evaluation settings using the public CUBS dataset, ensuring fair model-to-model comparisons.

Biswas et al. [15] presented a qualitative review contrasting traditional and neural network-based CIMT measurement techniques, highlighting the clinical potential of automation. Our work complements this by offering quantitative validation of segmentation accuracy and real-time performance metrics across U-Net variants. In addition, to facilitate reproducibility and future extensions, we have publicly released our source code and experimental configurations [31].

Lastly, Tian et al. [16] investigated U-Net family models for radial artery tracking in short-axis views. Their focus on arterial tracking differs from our emphasis on CIMT boundary segmentation, yet both studies underscore the importance of selecting suitable architectures based on anatomical and clinical contexts. Collectively, these distinctions highlight the unique contribution of our study as a systematic, reproducible analysis of U-Net variants for CIMT segmentation on a standardized public dataset.

Furthermore, our analysis of computational efficiency provides critical insights for the deployment of deep learning models on resource-constrained devices, such as portable or point-of-care ultrasound systems [32]. With the increasing adoption of handheld ultrasound devices in primary care and remote settings, there is a growing demand for lightweight algorithms that can operate with limited processing power and battery life [33]. While complex models like Attention U-Net offer peak performance, their high computational cost may hinder real-time deployment on mobile hardware. In contrast, our findings indicate that efficient architectures such as UNeXt achieve competitive accuracy with significantly lower latency, making them highly suitable for embedded systems where computational resources are limited. This trade-off is consistent with recent trends in mobile health AI, emphasizing the need to balance model complexity with accessibility and real-time capability [34].

A primary limitation of the present evaluation stems from the restricted scope of the CUBS dataset [28]. Our analysis was confined to the far-wall segments of the CCA, covering both the right and left arteries of the 1088 subjects. This inherent constraint limits the generalizability of the reported results to other anatomically and clinically critical segments of the carotid tree, such as the carotid bulb or the internal carotid artery, which are known to be preferential sites for plaque formation and pronounced thickening. Consequently, validation studies incorporating a wider range of carotid segments are necessary to assess the clinical utility of the evaluated automated methods for plaque characterization and stenosis grading.

Future research will focus on incorporating multi-observer consensus labels, addressing inter-observer variability, and extending our analysis to fully automated CIMT measurement pipelines integrated with end-to-end clinical decision support.

## 5. Conclusions

In this study, we performed a systematic and fair comparison of U-Net and nine of its widely used variants for automated CIMT segmentation in carotid B-mode ultrasound images. By training all models under identical conditions using a multi-center public database, we demonstrated the relative strengths and limitations of each architecture in terms of segmentation accuracy, boundary reconstruction characteristics, computational cost, and real-time applicability. Our findings confirmed that Attention U-Net achieved the highest Dice and IoU scores, whereas UNet++ provided notably smooth boundary reconstruction through its nested skip-connection design. In contrast, UNeXt showed competitive accuracy despite having the smallest parameter count and fastest inference time, implying strong suitability for real-time clinical applications or embedded ultrasound systems. Therefore, the selection of an appropriate CIMT segmentation model should consider both clinical requirements and system-level constraints, such as hardware availability and the need for real-time analysis. Overall, this work offers valuable reference information for both researchers and clinicians by highlighting performance trade-offs among state-of-the-art U-Net variants.

## Figures and Tables

**Table 1:** Key architectural characteristics of U-Net and its variants.

Model	Core Mechanism	Key Features	Filter Configuration
U-Net	CNN (Encoder–Decoder)	Skip connections, Double conv blocks	32, 64, 128, 256, 512
UNet++	Nested skip connections	Dense convolution blocks on skip paths	31, 62, 124, 248, 496
ResUNet	Residual learning	Residual blocks, Skip connections	32, 64, 128, 256, 512
AttentionUNet	Attention mechanism	Attention gates in skip connections	64, 128, 256, 512, 1024
AttentionResUNet	Residual + Attention	Residual blocks + Attention gates	33, 66, 132, 264, 528
SEUNet	Channel Attention	Squeeze-and-Excitation blocks	33, 66, 132, 264, 528
DenseUNet	Dense Connections	Dense blocks, Feature reuse	124, 376, 500, 748, 1244
InceptionUNet	Multi-scale Learning	Inception modules (1 × 1, 3 × 3, 5 × 5 convs)	68, 136, 272, 544, 1088
TransUNet	Transformer + CNN	Vision Transformer in encoder	CNN: 48, 96, 192, 384 Transformer: 1024
UNext	MLP + CNN	Tokenized MLP blocks in latent space	36, 72, 144

**Table 2:** Parameter counts per model.

Model	Number of Parameters
U-Net	7,845,489
UNet++	8,900,000
ResUNet	8,211,456
AttentionUNet	31,387,045
AttentionResUNet	8,710,000
SEUNet	8,519,112
DenseUNet	12,304,789
InceptionUNet	8,908,123
TransUNet	14,228,256
UNext	416,489

**Table 3:** Segmentation performance: Dice and IoU scores (Mean ± Standard Deviation).

Model	Dice Score	IoU Score
U-Net	0.8148 ± 0.0032	0.6882 ± 0.0030
UNet++	0.8198 ± 0.0024	0.6951 ± 0.0022
ResUNet	0.8103 ± 0.0024	0.6818 ± 0.0033
AttentionUNet	0.8216 ± 0.0034	0.6979 ± 0.0041
AttentionResUNet	0.8129 ± 0.0027	0.6857 ± 0.0033
SEUNet	0.8173 ± 0.0009	0.6919 ± 0.0014
DenseUNet	0.8124 ± 0.0043	0.6847 ± 0.0042
InceptionUNet	0.8094 ± 0.0048	0.6806 ± 0.0048
TransUNet	0.8143 ± 0.0044	0.6874 ± 0.0057
UNext	0.8051 ± 0.0052	0.6745 ± 0.0039

**Table 4:** Segmentation performance: Precision, Recall, and Accuracy (Mean ± Standard Deviation).

Model	Precision	Recall	Accuracy
U-Net	0.8687 ± 0.0184	0.7873 ± 0.0181	0.9818 ± 0.00014
UNet++	0.8704 ± 0.0041	0.7912 ± 0.0022	0.9821 ± 0.00005
ResUNet	0.8642 ± 0.0140	0.7827 ± 0.0036	0.9813 ± 0.00020
AttentionUNet	0.8194 ± 0.0204	0.8041 ± 0.0182	0.9947 ± 0.00017
AttentionResUNet	0.8662 ± 0.0096	0.7846 ± 0.0117	0.9816 ± 0.00020
SEUNet	0.8691 ± 0.0243	0.7895 ± 0.0246	0.9820 ± 0.00016
DenseUNet	0.8655 ± 0.0076	0.7843 ± 0.0063	0.9815 ± 0.00013
InceptionUNet	0.8636 ± 0.0094	0.7814 ± 0.0086	0.9812 ± 0.00012
TransUNet	0.8680 ± 0.0170	0.7867 ± 0.0125	0.9817 ± 0.00017
UNext	0.8601 ± 0.0206	0.7796 ± 0.0240	0.9811 ± 0.00064

**Table 5:** Comparison of computational time.

Model	Total Training Time (s)	Mean Epoch Time (s)	Inference Time (s)
U-Net	2007.23	40.14	0.0631
UNet++	2057.15	41.14	0.0632
ResUNet	2045.62	40.91	0.0632
AttentionUNet	3611.29	72.22	0.1033
AttentionResUNet	2042.60	40.85	0.0631
SEUNet	2034.11	40.68	0.0631
DenseUNet	2081.93	41.64	0.0633
InceptionUNet	1956.29	39.13	0.0629
TransUNet	2211.13	44.22	0.0682
UNext	1622.58	32.45	0.0598

## Data Availability

The original data presented in the study are openly available in https://github.com/JEongSJ-jsj/CIMT-Segmentation-Unet_variation (accessed on 15 December 2025).

## Abbreviations

The following abbreviations are used in this manuscript:

CIMT	Carotid intima-media thickness
CUBS	Carotid ultrasound boundary study
CCA	Common carotid artery
CNN	Convolutional neural network
IOU	Intersection over union
TP	True positives
FP	False positives
TN	True negatives
FN	False negatives
SE	Squeeze-and-excitation
CLAHE	Contrast-limited adaptive histogram equalization
GT	Ground Truth
